# Insulin post-transcriptional regulation via PARP12-mediated ADP- ribosylation

**DOI:** 10.21203/rs.3.rs-6473437/v1

**Published:** 2025-05-21

**Authors:** Soumyadeep Sarkar, Fangjia Li, Youngki You, Emily C. Elliott, Kevin J. Zemaitis, Hyeyoon Kim, Lye Meng Markillie, Jeremiah Traeger, Samantha M. Powell, Mireia Ramos-Rodriguez, Xiaoyan Yi, Jacob R. Enriquez, Marina Gritsenko, Hugh Mitchell, Decio L. Eizirik, Anthony K. L. Leung, Lorenzo Pasquali, Lori Sussel, Thomas O. Metz, Raghavendra G. Mirmira, Ernesto S. Nakayasu

## Abstract

ADP-ribosylation is a common modification that occurs in proteins and nucleic acids, regulating many cellular processes ranging from DNA repair to inflammatory signaling. ADP-ribosylation plays an important role in cancer biology, infectious diseases, and obesity, but its role in the development of type 1 diabetes is not well understood. Here, we studied the role of ADP-ribosyltransferase PARP12 in type 1 diabetes development. PARP12 expression is highly induced in human islets treated with pro-inflammatory cytokines or β cells from diabetic donors. Proteomics analysis of MIN6 insulin-producing cells identified that the RNA machinery is regulated by PARP12 during inflammation. PARP12 also ADP-ribosylates 150 mRNAs, including the insulin mRNA. This mRNA ADP-ribosylation in turn modifies transcript localization and halts translation. Overall, our data identified a role for PARP12 in ADP-ribosylation and translation halting of mRNAs, which may affect insulin production during insulitis.

## Introduction

Type 1 diabetes (T1D) is a devastating disease that reduces life expectancy by 11 years for men and 13 years for women [1]. The disease currently affects approximately 8.4 million people worldwide [2]. Treatment is lifelong and based on controlling the blood glucose levels with diet, exercise, and insulin administration. Despite advances in blood glucose control, the disease leads to complications such as cardiovascular and renal diseases, which account for the reduction in life expectancy. Mechanistically, T1D is an autoimmune disease with characteristic insulitis pathology [3] marked by infiltration of immune cells and apoptosis of insulin-producing pancreatic β cells mediated by pro-inflammatory cytokines and chemokines [4]. However, the highly complex cellular signaling transduced by the cytokines and chemokines is only partially characterized.

ADP-ribosylation plays a central role in regulating DNA repair, inflammatory signaling and anti-viral response [5], but its role in insulitis is not well understood. This modification is characterized by the addition of adenosine diphosphate (ADP)-ribose units to proteins and nucleic acid by ADP-ribosyltransferases (PARPs or ADRTs) with nicotinamide-adenosine dinucleotide (NAD) as a donor [6, 7]. ADP-ribosylation can also modify RNAs[8], but has only been characterized in bacteria, where it plays a role in antiviral response[9]. ADP-ribosylation occurs as single units (mono-ADP-ribosylation or MARylation) or chains (poly-ADP-ribosylation or PARylation) [7]. ADP-ribosylation is reversible and can be removed by hydrolases such as poly(ADP-ribose)glycohydrolase (PARG) and ADP-ribosylhydrolases [10]. In DNA repair, ADP-ribosylation labels damaged chromatin regions, recruiting the repair machinery. In immune cells, ADP-ribosylation enhances the activity of key transcription factors in inflammation, such as STATs, NF-κB, and NFATc3 [11–13]. Pro-inflammatory cytokines, such as IFN-γ, induce the expression of PARP9-14 which in turn regulate antiviral responses by targeting viral RNAs to degradation, viral replication, and protein translation [5].

Related to T1D, PARP1 deletion prevents the development of diabetes induced by streptozotocin in mice [14–16]. We have recently shown that the ADP-ribosylhydrolase ARH3, which removes ADP-ribosylation from serine residues [17], mediates the reduction of cytokine-induced apoptosis and production of chemokine CXCL9 by omega-3 fatty acids in MIN6 insulin-producing cells [18, 19]. Mechanistically, omega-3 fatty acids induce the degradation of SUZ12, a component of the histone methylation polycomb PCR2, leading to a reduction in histone methylation and increasing the expression of ARH3. However, it is unknown if the other 16 mammalian PARPs or 3 hydrolases have functions in T1D development.

Here, we investigated the role of PARP12 in insulitis owing to its expression profile in previous omics datasets [19–22]. We performed genomic analysis to determine mechanistically how the expression of PARP12 is regulated by pro-inflammatory cytokines in insulitis. We next performed a combination of RNA silencing and proteomics to determine possible functions of PARP12. This investigation identified PARP12 as an RNA ADP-ribosyltransferase, playing a role in post-transcriptional regulation of inflammation.

### Material and Methods

## Data reanalysis

Protein relative abundances (fold changes) of all 17 mouse and human PARPs were extracted along with their statistical significance from previously published proteomics datasets from our laboratory. The model systems included mouse insulinoma cell line MIN6 treated with cytokine cocktail CT1: IFN-γ, IL-1β and TNFα for 24 h [19], human islets treated with CT2: IFN-γ and IL-1β for 24 h [21], and human insulin-producing cell line EndoC-βH1 treated with CT2: IFN-γ and IL-1β for 48 h [22] or CT3: IFN-α for 8 h and 48 h [20] (**ESM Table S1**).

For single-cell RNA seq analysis, data from human islets were downloaded from the Human Pancreas Analysis Program (HPAP) portal (https://hpap.pmacs.upenn.edu/) and processed with Cell Ranger (v6.1.2) as previously described [23] (donor characteristics in **ESM Table S2**). After cell type annotation, we retrieved β cells from donors normoglycemic (non-diabetic, ND), positive for 1 (AAB1+) or multiple autoantibodies (AAB2+), and with T1D. In total, we analyzed 6,423 β cells from 15 ND, 5,751 from 9 AAB1+, 4,977 from 2 AAB2+, and 2,013 from 11 T1D donors. Differential gene expression analysis for β cells between T1D, AAB2 + and AAB1 + vs ND was performed using the *FindMarkers* function from Seurat, employing the Wilcoxon rank-sum test. P-values were adjusted using the Bonferroni correction method to account for multiple comparisons for all the genes (**ESM Table S3**).

Chromatin features (ATAC-seq and H3K27ac CHIP-seq) and gene expression (RNA-seq), along with annotated regulatory elements in human pancreatic islets treated or untreated with CT2 (IFN-γ and IL-1β) for 48 h, were obtained from our previous publication [22]. The datasets were lifted over from hg19 to hg38 genome assemblies. We selected regulatory elements within a 40 kb window centered on the PARP12 locus and divided them into three groups: Promoter/SRE (n = 1, PARP12 promoter), Distal/Opening_IRE (n = 2, increased accessibility and H3K27ac upon cytokines), and Distal/SRE (n = 7, without after cytokine exposure). Motif enrichment analysis was conducted using AME from the MEME suite (version 5.4.1) [24], testing for enrichment of cytokine-regulated TF motifs (i.e., transcription factors whose expression is upregulated upon cytokine exposure) across the three groups of regulatory elements, using shuffled sequences as a control.

### Cell culture

MIN6 cells were a gift from the Yamagata lab and were grown in DMEM containing 4.5 g/L each of D-glucose and L-glutamine, 10% FBS, 100 units/mL penicillin, 100 µg/mL streptomycin, and 50 mM 2-mercaptoethanol maintained at 37 ºC in a 5% $CO_2$ atmosphere. For PARP12 knockdown experiments, cells were treated at 80% confluency using Lipofectamine RNAiMAX (Invitrogen, Cat# 13778150) with SMARTpool ON-TARGETplus non-targeting siRNA (Dharmacon, cat#D-001810-10-20) or siRNA targeting *Parp12* (Dharmacon, cat# L-065127-01-0020), with concomitant treatment with CT2 (100 ng/mL IFN-γ: R&D, cat#485-MI-100, 10 ng/mL TNF-α: R&D, Cat#410-MT-010, and 5 ng/mL IL-1β: R&D, cat #401-ML-005) for 24 h. For pulse-chase experiments with stable isotope labeling by/with amino acids in cell culture (SILAC), cells were treated in DMEM containing heavy $^{13}C_6{}^{15}N_2$-lysine (Thermo Fisher Scientific, Cat# A33969).

### Label-free proteomics analysis

The MIN6 cell pellets were resuspended in 50 mM Tris-HCl, 8 M urea, and 10 mM dithiothreitol, and incubated for 1 h at 37 ºC with shaking at 800 rpm. Subsequently, 400 mM iodoacetamide was added to a final concentration of 40 mM, and the samples were incubated for 1 h in the dark at room temperature. The samples were then diluted 8-fold with 50 mM Tris-HCl, and 1 M $CaCl_2$ was added to a final concentration of 1 mM. Proteins were digested overnight at room temp using trypsin at an enzyme-to-protein ratio of 1:50. The digested peptides were desalted using C18 cartridges (Discovery, 50 mg, Sulpelco) and dried in a vacuum centrifuge. Peptides were analyzed using a Q Exactive Plus mass spectrometer (Thermo Scientific) as previously described[18]. The data were processed with MaxQuant software (v.1.5.5.) [25] using the mouse reference proteome database from UniProt Knowledge Base (downloaded on 02-18-2022, 17082 entries). Protein N-terminal acetylation and oxidation of methionine were set as variable modifications, and cysteine carbamidomethylation was set as a fixed modification. The software's default mass shift tolerance was used. Only fully tryptic-digested peptides were considered, with up to two missed cleavage sites allowed per peptide. Protein quantification was performed using the intensity-based absolute quantification (iBAQ) method [26, 27]. The data was normalized across samples based on total protein level, followed by substituting 1/4th of the lowest value from the dataset as missing values and log2 transformed. Statistically significant proteins ($p \leq 0.05$) were determined using Student's *t*-test. Network analysis of the statistically significant proteins was done with String database (V12.0) [28].

### Immunofluorescence imaging

MIN6 cells were cultured in 35 mm culture dishes (MatTek, Cat#P35G-1.5-10-C) and remained covered with PBS (Gibco, Cat#10010023) throughout the imaging process. They were permeabilized with 0.5% Triton X-100 for 30 min, followed by blocking with 1× PBS containing 5% goat serum and 0.3% Triton X-100 for 1 h at room temperature. The sections were incubated in 1:500 dilution of monoclonal rabbit anti-insulin IgG (Cell Signaling Technology, 3014), PARP12 (G bioscience, ITN2133-50u-555) and G3BP (Thermo Fisher, Cat#CL488-66486) overnight at 4 ºC. The primary antibody was thoroughly washed with PBS followed with incubation in 1:1000 dilution goat anti-rabbit IgG (H + L) highly cross-adsorbed secondary antibody, Alexa Fluor Plus 800 (Thermo Fisher Scientific).

For the fliFISH analysis, each primary FISH probe was composed of about 20 nucleotides complementary to the target mRNA and 28 nucleotides complementary to the secondary probe (Integrated DNA Technologies) [29] (**ESM table S4**). The properties of the probes, such as Tm, DG, and hybridization efficiency, were evaluated [30] and their targeting specificity was confirmed with BLAST (https://blast.ncbi.nlm.nih.gov/Blast.cgi). FISH probe solution was prepared by incubating 0.5 μM of each primary probe and 5 μM secondary probe (conjugated Alex 647 dye in both oligonucleotide termini) in 50 M Tris-HCl, pH 7.9, containing 100 M NaCl, 10 mM $MgCl_2$ and 1 mM dithiothreitol at 85 ºC for 3 min, followed by a gradual cool down to room temperature to hybridize the primary and secondary probes. Cell slides were immersed in 70% ethanol overnight at 4 ºC to permeabilize cell membranes. The slides were incubated with the FISH probe solution diluted to a final concentration of 4 nM of each probe in hybridization buffer (10% dextran sulfate, 2 mM vanadyl-ribonucleoside complex, 0.02% BSA, 2 × SSC, 10% formamide) overnight incubation at 37 ºC. Slides were thoroughly rinsed with buffer containing 2 × SSC and 10% formamide. The cell nuclei were stained with 0.05% DAPI.

Imaging was carried out using a Zeiss LSM 710 confocal laser scanning microscope, captured using a 100× oil immersion objective (NA 1.47). Laser excitation was set to 633 nm for *Ins2* (Alexa Fluor 647), 542 nm for PARP12 antibody (Alexa Fluor 555), and 405 nm for DAPI. Fluorescence emission was detected using spectral settings of 650–700 nm for Alexa Fluor 647, 570–620 nm for Alexa Fluor 555, and 420–480 nm for DAPI. Key imaging parameters, including detector gain, laser power, and pinhole size, were optimized to minimize photobleaching while ensuring a high signal-to-noise ratio for each channel. Image processing was performed using FIJI/ImageJ, applying only linear adjustments to brightness and contrast without modifying pixel intensity values.

### Prediction of RNA binding to PARP12 zinc finger motifs

The interaction strength of *Ins2* pre-mRNA with PARP12 was calculated using the RPISeq program (http://pridb.gdcb.iastate.edu/RPISeq/index.html). For PARP12, we used sequences from three zinc finger (ZnF1-3) domains in the following combinations: ZnF1 and 2, ZnF2 and 3, and ZnF1, 2 and 3. The interaction strength was predicted in 50-nucleotide segments of *Ins2* pre-mRNA with 10-nucleotide overlapping in both ends.

## Prediction of the PARP12 structure

The PARP12 structure predicted with AlphaFold (identifier AF-Q8BZ20-F1) was downloaded from Uniprot Knowledgebase and analyzed with Discovery Studio Visualizer v21. The *Ins2* linear RNA oligonucleotide (ACAGGCAUGCAACCCCUGCCACCUG) with the highest predicted binding was positioned on PARP12, based on the ZnF motif orientations similar to RNA-bound protein structures of PDB, 3D2S and 7C07. The geometry of the RNA-bound PARP12 model was optimized using Discovery Studio Visualizer.

### ADP-ribosylated RNA pulldown

MIN6 cells were harvested using Tri reagent (Zymo Research, R2050-1-200) and the RNA was extracted using RNA Clean & Concentrator™-5 kit (Zymo Research, R1014), following manufacturer instructions. To test the specificity of the ADP-ribosylation-binding AF1521 macrodomain, an RNA aliquot was pre-treated with 2 nM SARS-CoV2 macrodomain MAC1 at room temp for 1 h in hydrolase reaction buffer (250 mM HEPES, 750 mM NaCl, 10 mM β-mercaptoethanol, 0.05 mM Triton X100, 5 mM $MgCl_2$, 1X RNAsecure) to remove ADP-ribosylation. Equal amount of total RNA from each sample was incubated with either 20 μL of Af1521 macrodomain conjugated to magnetic beads (Tulip Biolabs, 2426) or control (naked or mutated Af1521) beads in 600 μL RNA pulldown buffer (10 mM Tris-HCl, 0.6 M NaCl, 0.1% NP40, 1X RNA secure) overnight at 4°C. After overnight incubation, the beads were gently washed with RNA pulldown buffer three times, and 50 μL of Tri reagent was added to elute RNA from the beads, followed by RNA Clean & Concentrator™-5 extraction.

### Quantitative real-time PCR analysis

Extracted total and Af1521-enriched RNAs were quantified using nanodrop and expression level of genes were measured in StepOnePlus RTPCR systems using QuantiNova™ SYBR Green RT-PCR reagent (Qiagen, Cat. No. / ID: 208154). Pre-designed mouse primers were ordered from Millipore sigma (Cat# KSPQ12012) and IDT (**EMS Table S5**). The average CT value of two housekeeping genes Rlp13 and Nono was used to calculate the fold change of genes using Livak's method [18, 31].

## RNAseq analysis

RNAs eluted post-incubation with AF1521 and Control (mutant AF1521) beads were processed for cDNA synthesis using SMARTer® Universal Low Input RNA Kit (Takara, cat#634938) according to instruction manual, followed by template library prep using NEBNext® Ultra™ II FS DNA Library Prep Kit for Illumina (NEB, cat#E7805S). Single-read sequencing of the barcoded cDNA libraries with a read length of 150 were performed on NextSeq 550 plateform using NextSeq500/550 High Output v2.5 150 cycles (cat#20024907). Quality control of RNA-seq data was performed using FastQC (Version 0.12.0) (https://www.bioinformatics.babraham.ac.uk/projects/fastqc/). Read trimming was performed using BBDuk (https://github.com/BioInfoTools/BBMap/blob/master/sh/bbduk.sh). Reads were aligned to the mouse genome GRCm39 using STAR[32] and mapped to genome features using the featureCounts program from the subread package (https://subread.sourceforge.net/). To account for technical variability raw counts were batch corrected with ComBat-Seq[33] with default parameters prior to differential expression analysis with DESeq2[34].

## Heavy isotope-labeled lysine proteomics

PARP12 siRNA cells were treated with CT1 in heavy isotope-labeled lysine SILAC media for 24 h. Samples were submitted to proteomics analysis as described above with the exception that the digestion was done with endoproteinase LysC instead trypsin. Raw files were processed in MaxQuant (v.2.5.1.0), using mouse reference protein datasets downloaded from UniprotKB (2023-03-01, 21,949 entries). The multiplicity was set to 2 considering light and heavy $^{13}C_6{}^{15}N_2$-lysine labels. Searches were performed with oxidation of methionine and protein N-terminal acetylation as variable modifications and cysteine carbamidomethylation as fixed modification. The quantification parameters included re-quantification and matching between runs with a matching and aligning time windows of 1 and 20 min, respectively. All remaining parameters were kept as the default settings.

## Intact insulin analysis by mass spectrometry

Pellets of 300,000 cells pulsed-chased in SILAC media were lysed in 10 µL of water. Aliquots of 2 µL were spotted onto poly-lysine coated indium-tin oxide slides (Bruker Daltonics, Billerica, MA). After drying, slides were serially washed for 30 s each with 70% ethanol, 100% ethanol, and 50 mM ammonium acetate. The spotted cells were prepared by spraying 5% acetic acid in 50% ethanol and subsequently applying 15 mg/mL 2,5-dihydroxyacetophenone as matrix in 90% acetonitrile with 0.2% trifluoroacetic acid using an M5 sprayer (HTX Technologies, Chapel Hill, NC)[35]. Slides were analyzed on an orbitrap mass spectrometer (Q Exactive HF, Thermo Fisher Scientific, Bremen, DE) retrofit with ultrahigh mass range (UHMR) boards and an EP-MALDI source (Spectroglyph LLC, Kennewick, WA). Briefly, the instrument was operated under custom privileges licenses, and mass spectra were collected with transients of 1.024 s (480k mass resolution at *m/z* 200) and a *m/z* range of 2000 to 20000. The raw data and position files were imported into SCiLS Lab (v.2021c; Bruker Daltonics, Bremen, DE) using automatic import and processing settings. Maximum intensity values were extracted with root mean square normalization across conditions.

## Data visualization and statistical analysis

Microsoft Excel was used to perform basic data analysis with GraphPad Prism 9 (Version 9.4.1) was used for statistical analysis and data visualization. Perseus-Max-Quant was used to create Heatmaps. Pathway analysis for Figs. 5 and 6 was performed using the core analysis setting of Ingenuity Pathway Analysis software using the Reactome database.

### Results

*PARP12 regulation by cytokines in islets and beta cells.*

To investigate the possible roles of PARPs in insulitis, we re-analyzed published proteomics data from human islets, EndoC-βH1 cells and MIN6 cells treated with different combination of pro-inflammatory cytokines (CT1: IFN-γ, IL-1β and TNFα; CT2: IFN-γ and IL-1β; CT3: INFα)[19–22]. This re-analysis revealed that ADP-ribosyltransferases PARP9, 10, 12, and 14 are consistently upregulated by the pro-inflammatory cytokines in all 5 datasets (Fig. 1A). We investigated the expression of PARPs in β cells from single-cell RNAseq data from donors at different stages of T1D development, determined by the number of detected islet autoantibodies and disease onset. The expression levels of *Parps* 1, 4, 5B, 6, 7, 8, 9, 11, 12, and 14 were significantly higher in β cells of individuals with T1D than those with islet autoantibodies compared to the absence of diabetes or non-diabetic donors (Fig. 1B). Together, the results showed that the expression levels of PARP9, 10, 12, and 14 were increased both *in vitro* models and donors with T1D. Notably, *Parp12* abundance was increased in the phospholipase *Pla2g6* knockdown MIN6 cells[18]. Further, *Adprhl2* (ARH3 protein) knockdown causes 52.3% reduction in PARP12 expression by the cytokine cocktail [18]. ARH3 expression is regulated by *Pla2g6* via the release of omega-3 fatty acids [18]. To test if PARP12 is also regulated by omega-3 fatty acids, we treated MIN6 cells with cytokine cocktail CT1 in the presence of the omega-3 fatty acids, eicosapentaenoic acid (EPA) and docosahexaenoic acid (DHA). The EPA and DHA increased the cytokine-induced *Parp12* expression by 54% and 62%, respectively (Fig. 1C). Based on these results, we hypothesized that PARP12 has a role in cytokine-mediated signaling and β-cell stress and selected it for further characterization.

To investigate the regulation of the gene encoding PARP12, we examined our previously published histone H3K27Ac enrichment profiles, RNA-seq and ATAC-seq data of CT2-treated human islets[22]. We detected a cytokine-induced enrichment of the active histone modification H3K27Ac upstream of the *PARP12* promoter region (Fig. 1D). The higher H3K27Ac levels matched with an increased chromatin accessibility measured by ATAC-seq (Fig. 1D). As expected, these cytokine-induced chromatin changes correlated with increased transcription activity, measured by RNA-seq data (Fig. 1D). To determine potential transcription factors involved in regulation of *Parp12* gene expression, we studied the sequence composition of the gene locus. We found that the *Parp12* gene promoter and distal regulatory elements bear binding motifs for POU2F2, JUNB, NFKB1, IRF1, IRF2, IRF4, IRF8, and IRF9 (Fig. 1E). Among those, IRF9 binding motif sequence in the promoter region of the gene was apparent, prompting functional analyses. Knockdown of *Irf9* in MIN6 cells reduced the cytokine cocktail CT1-mediated induction of *Parp12* by 52% (Fig. 1F), confirming the role of this transcription factor in regulating *Parp12* gene expression.

Overall, these data identified PARP12 as a cytokine-regulated PARP during insulitis in islets and that its gene expression is regulated by IRF9.

## Proteomics of PARP12 knockdown MIN6 cells

To gain knowledge on PARP12 function, we performed proteomic analysis of cytokine cocktail CT1-treated MIN6 cells with or without *Parp12*-targeting siRNA. As a control, we observed 62% and 85% knockdown efficiency of both *Parp12* mRNA and its encoded protein levels in the CT1-treated group, respectively (Fig. 2A). Our label-free proteomic analysis identified 4898 proteins, of which 683 proteins were regulated by the cytokine cocktail (Fig. 2B, **ESM Table S6**). Moreover, 63 of the cytokine-regulated proteins were significantly affected by PARP12 (Fig. 2C), which were submitted to a network analysis. The network analysis identified a cluster of interacting proteins related to transcription and post-transcription regulation: IRGM1, IRF9, PARP10, ZC3HAV1 and OAS3 (Fig. 2D). In the cytokine-treated groups, the production of these proteins was partially or completely dependent on PARP12 (Fig. 2E). Together, the proteomics analysis indicates that PARP12 regulates proteins related to transcription and post-transcription regulation.

## PARP12 post-transcriptionally regulates insulin 2

To investigate the possibility of PARP12 being a post-transcriptional regulator, we analyzed the proteins that were affected by the knockdown of *Parp12* siRNA in cells treated with CT1. We found 94 proteins with a significant increase in cells treated with *Parp12* siRNA and CT1 compared to parental cells treated with CT1 (Fig. 3A). Insulin 2 (INS2) was one of the proteins whose abundance was downregulated by PARP12 (Fig. 3B). However, its mRNA level was not significantly altered (Fig. 3C), supporting that PARP12 post-transcriptionally regulates insulin production. We next performed *in situ* hybridization of *Insulin 2 (Ins2)* mRNA in MIN6 parental and PARP12 knockdown cells treated with cytokines. The cells were also immunostained for PARP12, which showed a perinuclear localization and increased abundance in cells treated with cytokines (Fig. 3D). As expected, *Parp12* siRNA reduced its protein abundance in both parental and siRNA cells (Fig. 3D). Under cytokine treatment, *Ins2* mRNA distribution moves from the perinuclear region and became diffused in the cytosol (Fig. 3D). In *Parp12* siRNA cells, *Ins2* mRNA remained in perinuclear localization, showing that its release into the cytosol is dependent on PARP12 (Fig. 3D). Overall, these data showed that PARP12 is a post-transcriptional regulator that releases the *Ins2* mRNA from the perinuclear region to a diffused distribution in the cytosol.

## PARP12 is a potential RNA ADP-ribosyltransferase

As PARP12 is an ADP-ribosyltransferase, we hypothesized that PARP12 ADP-ribosylates the *Ins2* mRNA to downregulate its translation. To investigate this possibility, we examined the PARP12 structures, including its catalytic domain (PDB ID: 6V3W) and an AlphaFold prediction (identifier AF-Q8BZ20-F1) of the remaining protein composed of two WWE (tryptophan-tryptophan-glutamate) domains, and four zinc finger domains (ZnF) (Fig. 4A). We assembled the *Ins2* mRNA onto this predicted structure based on the catalytic site and the ZnF domains of PARP12. These four ZnF domains are predicted to form a groove that could accommodate RNA molecules (Fig. 4A). Of these four, ZnF motifs 1, 2 and 3 are predicted to bind to the RNA and were therefore used to identify possible *Ins2* mRNA regions that might interact with PARP12. The *Ins2* mRNA was divided into 27 oligonucleotide sequences of 50 nucleotides with 10 overlapping nucleotides between oligonucleotides. The oligonucleotide with the highest prediction corresponded to a region in intron 2 of insulin 2 mRNA (Fig. 4B). We then positioned the oligonucleotide with the highest predicted binding affinity onto the PARP12 structure. This prediction analysis showed that the PARP12 structure can accommodate RNA molecules in proximity to its catalytic site (Fig. 4A), indicating this enzyme may ADP-ribosylate transcripts such as *Ins2*.

To determine if the *Ins2* mRNA gets ADP-ribosylated, we extracted RNAs from MIN6 cells treated or not with CT1 followed by pull-down of the ADP-ribosylated RNAs with AF1521 ADP-ribosylation binding macrodomain and performed qPCR (Fig. 4C). *Ins2* mRNA was amplified in both untreated and CT1-treated cells (Fig. 4D). As a control, pulldowns with beads alone without AF1521 showed only trace amplification (Fig. 4D). To test the specificity of AF1521 beads, we pretreated RNA aliquots with SARS-CoV2 macrodomain MAC1 to remove ADP-ribosylation (Fig. 4C). This treatment led to a 94–99% reduction in *Ins2* mRNA amplification (Fig. 4D), confirming that the AF1521 specifically enriches for ADP-ribosylated RNAs. Together, these data show that PARP12 is a potential RNA ADP-ribosyltransferase and that the *Ins2* mRNA is ADP-ribosylated.

## Landscape of PARP12 ADP-ribosylated RNAs

To determine if PARP12 is an RNA ADP-ribosyltransferase and to identify its substrates, we enriched ADP-ribosylated RNAs from PARP12 knockdown cells treated with cytokines CT1 with AF1521 beads and submitted them to RNAseq. By quantifying RNAs enriched in AF1521 vs. control beads, 1381 RNAs were found to be ADP-ribosylated. From these, 150 RNAs were significantly downregulated in PARP12 knockdown cells (Fig. 5A, **ESM table S7**), therefore are potential PARP12 substrates. A function enrichment analysis of the PARP12 substrates showed top enrichment of genes related to respiratory electron transport, cellular responses to stimuli, metabolism of proteins and RNAs, immune system and cell cycle (Fig. 5B, **ESM table S8**). PARP12 knockdown led to a 63% reduction in insulin 2 ADP-ribosylation in cells treated with CT1 but no significant changes were observed in untreated cells (Fig. 5C). These results show that ADP-ribosylation is a major RNA modification and that PARP12 is an RNA ADP-ribosyltransferase that modifies many transcripts, including the insulin 2 mRNA.

## PARP12 suppresses the translation of specific proteins

We next tested if PARP12-mediated ADP-ribosylation of RNAs has a role in regulating protein translation. We treated PARP12 siRNA MIN6 cells with CT1 and pulse-chased them with heavy isotope-labeled lysine for 24 h to label nascent proteins and analyzed by proteomics. The analysis identified 187 proteins whose translation was affected by PARP12 in untreated and CT1-treated cells (Fig. 6A, **ESM table S9**). A functional-enrichment analysis revealed that 19 pathways categories, with top enriched pathways from Cellular response to stimuli, metabolism of protein and RNA, were influenced by PARP12 (Fig. 6B, **ESM table S10**). As the proteomics analysis did not yield to detectable insulin peptides, we measured insulin 2 by intact protein mass spectrometry analysis. Insulin 2 translation is repressed by the CT1 treatment, but this repression is alleviated in *Parp12* siRNA cells (Fig. 6C). These results show that PARP12 regulates the translation of many proteins, including repressing insulin 2 production.

### Discussion

Our data showed that PARP12 expression is induced by pro-inflammatory cytokines in human islets and insulin-producing cell lines. PARP12 is also elevated in β cells during T1D development. Mechanistically, cytokines induce PARP12 gene expression by histone acetylation, chromatin opening and activation of the transcription factor IRF9. IRF9 activated by interferon signaling, has an essential role in the expression of interferon-stimulated genes (ISGs) during antiviral responses [36]. Indeed, PARP12 is an antiviral factor that suppresses Zika virus by targeting its non-structural proteins NS1 and NS3 to proteasomal degradation [37]. PARP12 also impedes viral replication by targeting viral RNAs in stress granules, protein-RNA aggregates, stalling the translation [38–40].

Here, we show that PARP12 blocks insulin 2 translation by ADP-ribosylating and translocating its transcript from the perinuclear region to the cytosol under cytokine influence. PARP12 overexpression causes global translational repression by associating with cellular translational machinery and potentially targeting mRNA to stress granules [39]. *E. coli* has a similar machinery that ADP-ribosylates and halts the translation of phage transcripts as part of an antiviral response [9]. Conversely, coronavirus ADP-ribosylhydrolase MAC1 counteracts the protective effect of PARP12[41]. At least under *in vitro* conditions, we show that MAC1 efficiently cleaves PARP12-mediated mRNA ADP-ribosylation, suggesting a counteracting mechanism for ADP-ribosylation-mediated translation halting. Our data show that a large portion of the cell transcriptome is ADP-ribosylated, but only a fraction is mediated by PARP12. Other mammalian PARPs ADP-ribosylate RNAs, but specific substrates remained unknown [8, 42]. Therefore, it is possible that the ADP-ribosylation of mRNAs is a much broader translation regulation mechanism. Besides targeting mRNAs, ADP-ribosylation represses translation by PARP16-mediated ADP-ribosylation of ribosomal protein subunits [43].

Our structural analysis predicts that PARP12 has four ZnF motifs that form a groove that accommodates the RNA chain. ZnF motifs can bind to DNA, RNA and ADP-ribosylation [44]. Similar ZnF motifs are also present in PARP13, also known as zinc finger antiviral protein (ZAP). Despite not displaying ADP-ribosylation activity, PARP13 binds to GC motifs in viral RNAs and target them to degradation [37]. Indeed, the insulin 2 mRNA region bearing three GC motifs was predicted to have the highest binding to PARP12, suggesting a possible similar binding specificity compared to PARP13. The structural analysis also identified two ADP-ribosylation-binding WWE domains, which are present in other PARPs and some E3 ubiquitin ligases [37]. PARP12 WWE domains help its recruitment to stress granules via poly-ADP-ribose signaling catalyzed by PARP1 [38]. Combined these structural insights align with our observation that PARP12 acts as a translation inhibitor under pro-inflammatory conditions, potentially binding RNA with its ZnF motifs and utilizing WWE domains for cellular localization and function.

The reduction in insulin production by PARP12 might aggravate insulin availability during T1D development. At this stage, pro-inflammatory cytokines such as interferons, which induce PARP12 expression, are major regulators of the insulitis process [45]. Our findings might also have potential implications in aggravation of infection in type 2 diabetic patients, who are already at higher risk for complications. Notably, SARS-CoV-2 infections are associated with a robust production of pro-inflammatory cytokines and insulin depletion [46, 47]. It is possible that the insulin depletion is caused, at least in part, by ADP-ribosylation of its mRNA and consequent block in translation. However, the involvement of PARP12 in this process still needs to be investigated.

In conclusion, we identified PARP12 as a cytokine-induced RNA ADP-ribosyltransferase that halts protein translation. Specifically, PARP12 targets insulin mRNA and ADP-ribosylating it and thereby reducing insulin production. This mechanistic insight underpins a potential physiological impact during T1D development, where reduced insulin availability can exacerbate the disease progression. Moreover, our findings offer a broader perspective on the role of PARP12 in inflammatory and antiviral responses, suggesting its involvement in the complex interplay between cytokine signaling and translational control.

## Declarations

#### Data availability

The proteomics data were deposited into the MassIVE repository, a member of the ProteomeXchange, under accession numbers/user MSV000097349 and MSV000097350 (accession information below). The ADP-ribosylated RNA sequencing data is available at NCBI Geo under accession number GSE292484.

Title: Insulin post-transcriptional regulation via PARP12-mediated ADP-ribosylation (global label-free)

MassIVE accession: MSV000097349

https://massive.ucsd.edu/ProteoSAFe/dataset.jsp?task=93584fb8b1df4ad8b5f998c7fafb4ab0

For now, manuscript reviewers can access the data via FTP using:

Server: massive.ucsd.edu

User: MSV000097349

Password: Insulin6433

Title: Insulin post-transcriptional regulation via PARP12-mediated ADP-ribosylation (pulse-chase SILAC)

MassIVE accession: MSV000097350

https://massive.ucsd.edu/ProteoSAFe/dataset.jsp?task=778b2027fd574d039b0ecd293259e851

Manuscript reviewers can access the data via FTP using:

Server: massive.ucsd.edu

User: MSV000097350

Password: Insulin6432

#### Competing interests

The authors declare that they have no competing interests.

#### Acknowledgments

The authors would like to thank the technical assistance and support of Mr. Armando Puente (University of Chicago), Dr. Mikhail Belov (Spectroglyph, LLC), Mr. Gordon Anderson and Mr. Chris Anderson (GAA Custom Electronics, LLC), Drs. Kyle Fort, Maria Reinhardt-Szyba, and Alexander Makarov (Thermo Fisher Scientific). Part of the work was performed in the Environmental Molecular Sciences Laboratory, a U.S. DOE national scientific user facility at Pacific Northwest National Laboratory (PNNL) in Richland, WA. Battelle operates PNNL for the US Department of Energy under contract DE-AC05-76RLO01830.

#### Author Contributions Statement

Conceptualization: SS, ESN. Investigation, data curation, formal analysis and validation: all authors. Visualization: SS, FL, YY, HK, MR-R, XY. Methodology: SS, FL, YY, KJZ, LMM, HK, AKL, RGM, ESN. Project administration, supervision and resources: DLE, AKLL, LP, LS, TOM, RGM, ESN. Funding acquisition: DLE, MR-R, LP, LS, TOM, RGM, ESN. Writing – original draft: SS, FL, YY, KJZ, HK, MR-R, XY, JRE, HM, ESN. Writing – review & editing: all authors.

#### Funding

This work was supported by the Catalyst Award from the Human Islet Research Network (HIRN) (to E.S.N) (via U24 DK104162) and by National Institutes of Health, National Institute of Diabetes and Digestive and Kidney Diseases (NIDDK) grants R01 DK138335 (to ESN, and TOM), U01 DK127505 (to LS and ESN), U01 DK127786 (to RGM, DLE, and TOM), R01 DK060581 (to RGM), R01 DK105588 (to RGM), and P30 DK020595 (to RGM). The development of ADP-ribosylated RNA sequencing assay was conducted under the Laboratory Directed Research and Development Program at PNNL (to ESN). This work was also supported by Spanish Ministry of Science and Innovation PID2023-151556OB-I00, CNS2024-154742, “la Caixa” Foundation, LCF-PR-HR24-00150 (to LP) and the 2024 EFSD/Lilly Young Investigator Research Award (MR-R).

## Figures

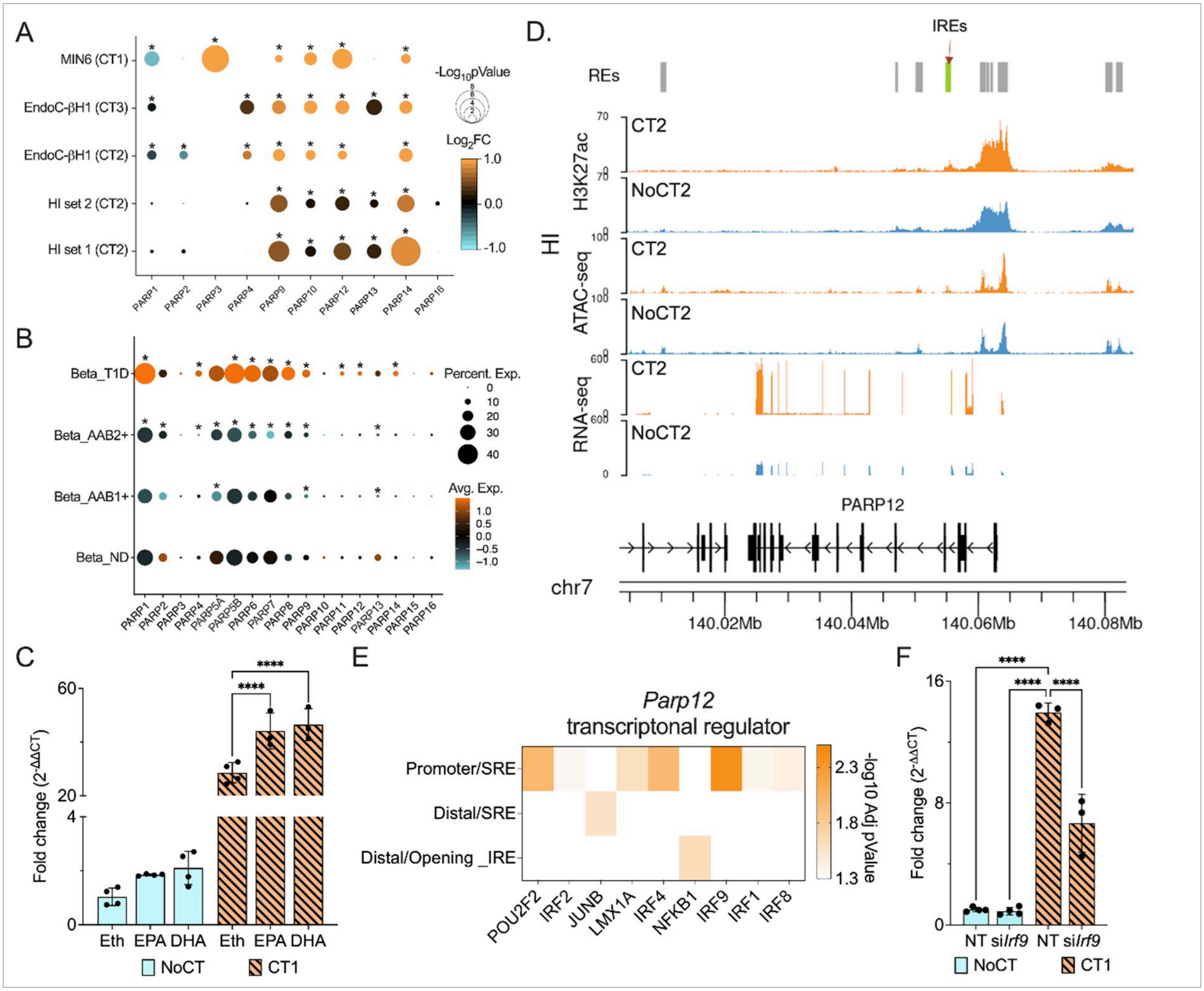

### Figure 1

Expression analysis of PARP12. (**A**) Quantification of PARP protein abundances in proteomics re-analysis of human islets[21], EndoC-βH1 cells[20, 22] and MIN6 cells[18] treated with combinations of pro-inflammatory cytokines: (CT1: IFN-γ, IL-1β and TNFα; CT2: IFN-γ and IL-1β; CT3: INFα). Asterisks represent significant expression of the respective protein compared to no cytokine-treated control. (**B**) Quantification of PARP gene expression in β cells in single-cell RNA-seq data downloaded from the Human Pancreas Analysis Program (HPAP) data portal (https://hpap.pmacs.upenn.edu/). The data were collected from pancreata from non-diabetic donors or donors at different stages of type 1 diabetes development: AAB1+ - individuals seropositive for 1 islet autoantibody, AAB2+ - individuals seropositive for multiple islet autoantibodies, beta – β cells, T1D – type 1 diabetes. We analyzed 6,423 β cells from 15 ND, 5,751 β cells from 9 AAB1+, 4,977 β cells from 2 AAB2+, and 2,013 β cells from 11 T1D donors. Asterisks represent significant gene expression compared to non-diabetic control. (**C**) Effect of omega-3 fatty acids, eicosapentaenoic acid (EPA) and docosahexaenoic acid (DHA), on the expression of *Parp12* gene induced by CT1 in MIN6 cells (n=3-4, 2way ANOVA, Holm-Šídák's multiple comparisons test). (**D**) View of the PARP12 locus regulation in human islets treated with CT2, depicted by histone acetylation (H3K27Ac) ChIP-seq, ATAC-seq and RNA-seq data[22]. (**E**) Genomic motif analysis of putative transcription factors that regulate PARP12 expression. (**F**) Regulation of *Parp12* gene expression by the transcription factor IRF9. *Parp12* mRNA level in MIN6 cells treated with CT1 in combination with NT (Non-target), *Irf9* siRNA (si*Irf9*) (n=4, 2way ANOVA, Holm-Šídák's multiple comparisons test). Outlier data points were identified and removed using Grubb's test for single outliers with p<0.3.

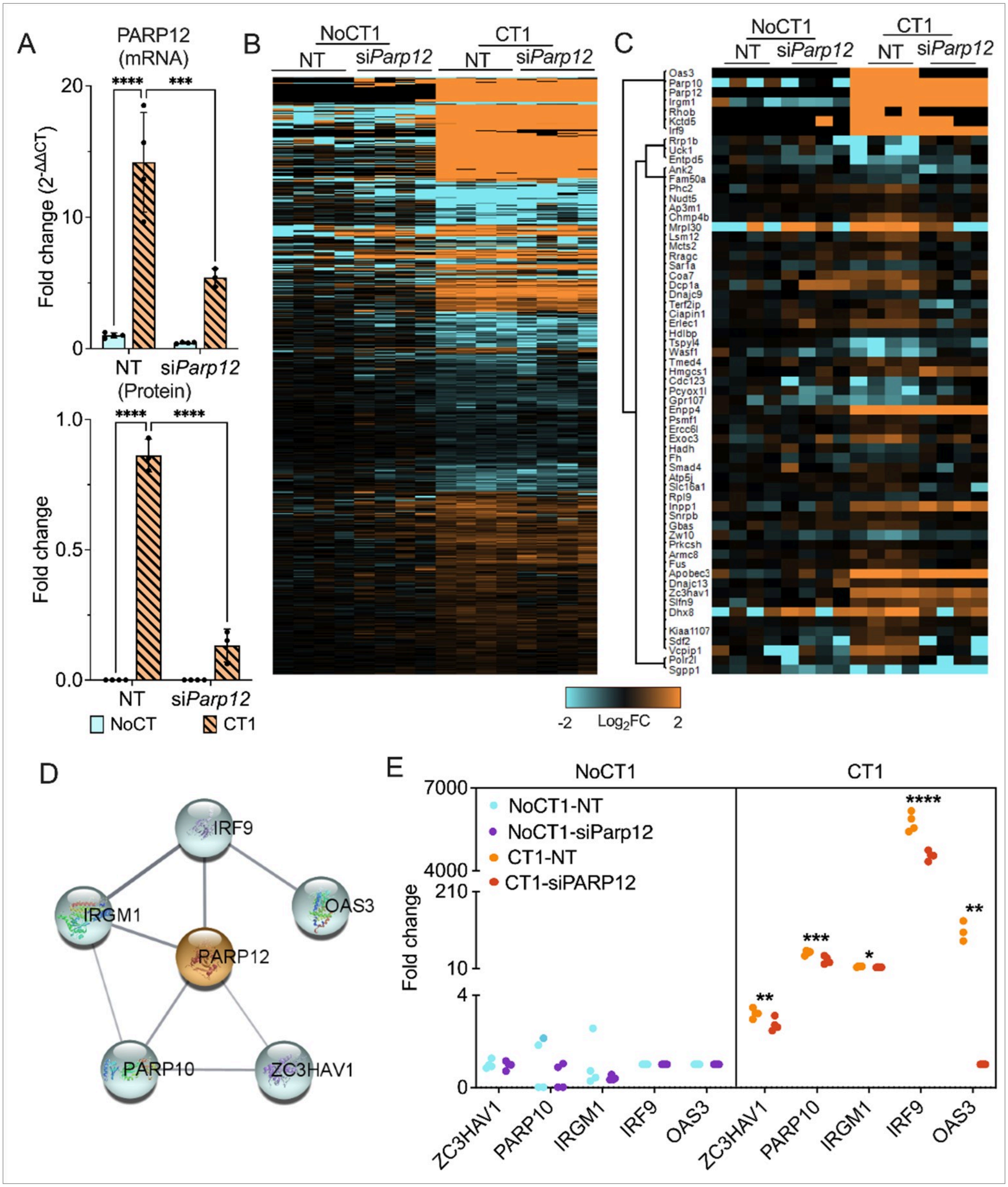

### Figure 2

Proteomics analysis of MIN6 cells exposed to Parp12 siRNA (siParp12) and treated with the pro-inflammatory cytokine cocktail CT1 (IFN-γ, IL-1β and TNFα). (**A**) PARP12 expression analysis in *Parp12* siRNA MIN6 cells treated with CT1. Samples were measured by qPCR (Top panel) and proteomics analyses (Bottom panel) (n=4, 2way ANOVA, Holm-Šídák's multiple comparisons test). (**B**) Heatmap representing changes in the global proteome of *Parp12* siRNA MIN6 treated with CT1 (n=4, Student's *t*-

test). (**C**) Subset of the heatmap of panel B that proteins are regulated by both CT1 and *Parp12* siRNA. (**D**) A network by the String database of proteins found in panel C. (**E**) Abundance profile of proteins in the network of panel D (n=4, 2way ANOVA, Holm-Šídák's multiple comparisons test). Outlier data points were identified and removed using Grubb's test for single outliers with p<0.3.

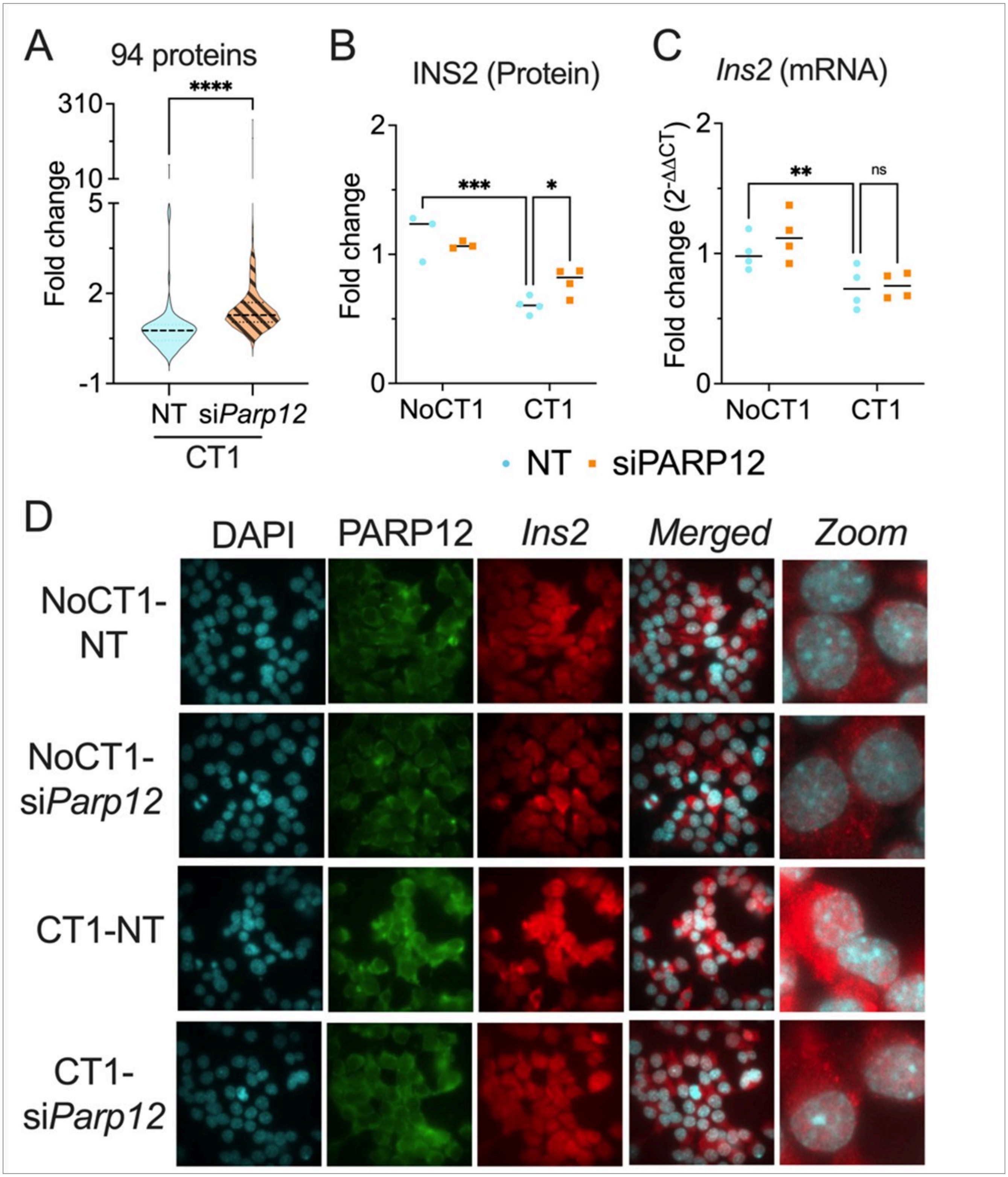

**Figure 3**

Regulation of insulin 2 by PARP12. (**A**) Abundance of 94 proteins regulated by PARP12 in MIN6 cells treated with the pro-inflammatory cytokine cocktail CT1 (IFN-γ, IL-1β and TNFα) and measured in the proteomics analysis (Mann-Whitney test). (**B**) Abundance profile of insulin 2 protein in the proteomics analysis (n=3-4, 2way ANOVA, Holm-Šídák's multiple comparisons test) (**C**) qPCR quantification of *Ins2* mRNA in *Parp12* siRNA MIN6 cells treated with CT1 (n=4, 2way ANOVA, Holm-Šídák's multiple comparisons test). (**D**) Fluorescence imaging analysis of *Ins2*mRNA (red) in *Parp12* siRNA MIN6 cells treated with CT1. In addition to Ins2 mRNA, PARP12 protein was stained by immunofluorescence (green) and nuclei with DAPI (cyan).

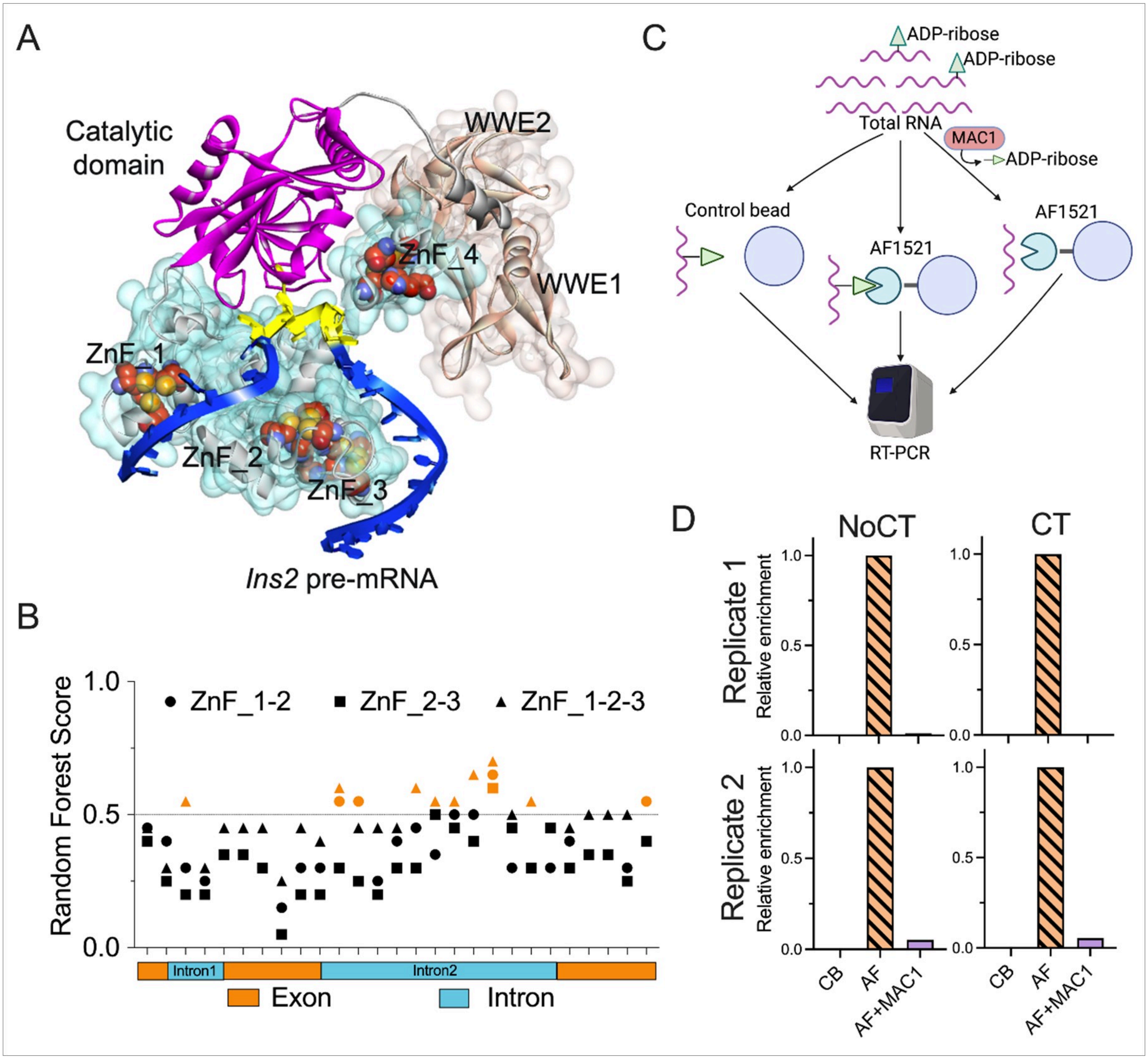

**Figure 4**

RT-PCR amplified *Ins2* intron2 pre-mRNA from MIN6 cells treated with -/+ si*Parp12* in CT1 treated group. (**A**) Predictive complex structure of PARP12 and *Ins2* mRNA. Four zinc finger motifs of PARP12 are highlighted by Corey–Pauling–Koltun representation. The yellow section of mRNA represents the potential ADPribosylation site as near the catalytic domain of PARP12. (**B**) The prediction of RNA-protein interactions was conducted using RPISeq. The random forest values for 27 segments of the *Ins2*pre-mRNA were calculated against three different combinations of zinc finger domains of PARP12. Predictions with probabilities greater than 0.5 were considered positive. (**C**) Scheme representing ADP-ribosylated RNA pulldown experimental design. (**D**) ADP-ribosylated RNA with/without MAC1 treatment were pulldown using AF1521 (AF) or naked-control beads (CB). Amplification was calculated against 0.3% Input and represented as relative enrichment to AF.

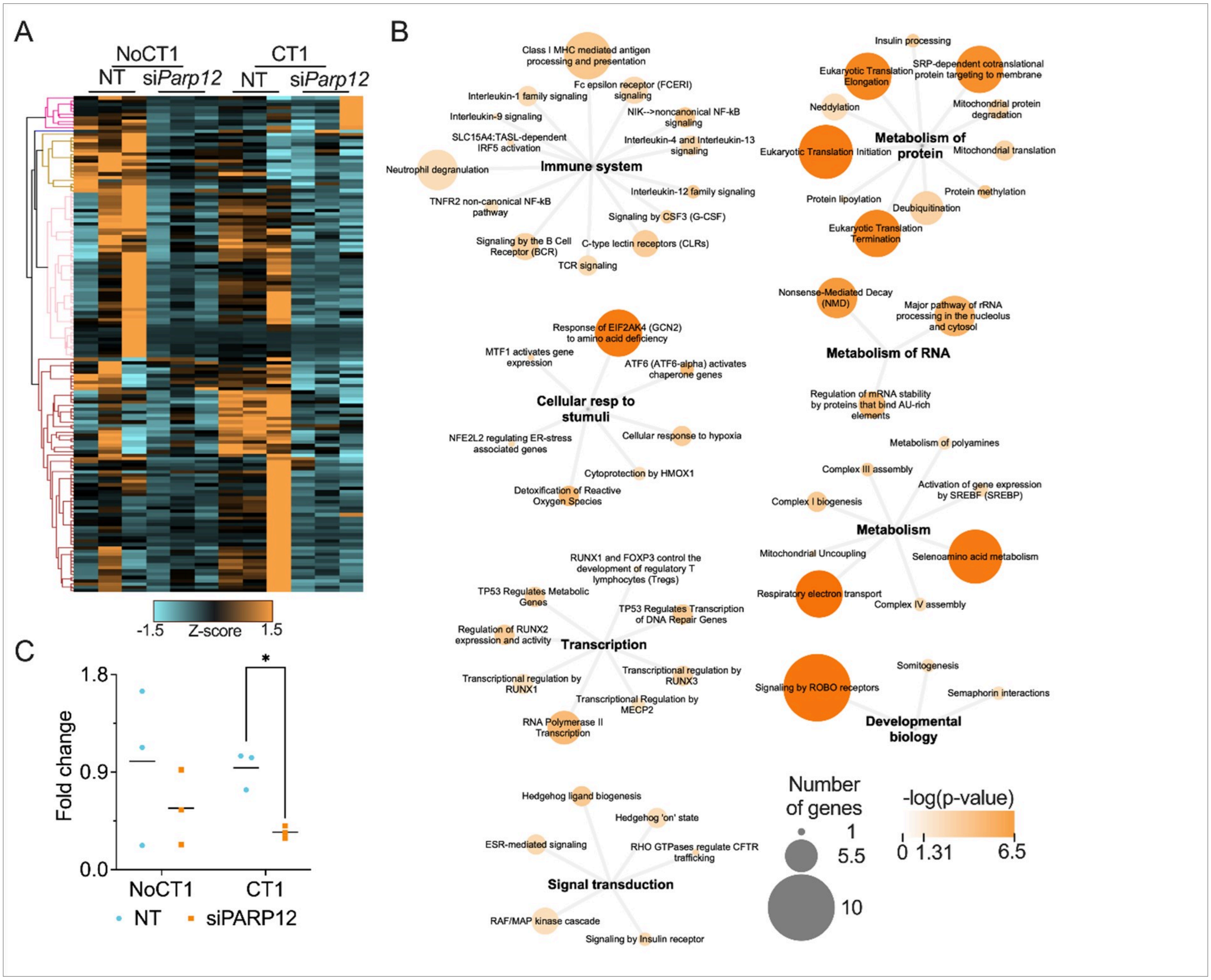

### Figure 5

PARP12 RNA substrates. (**A**) Heat map representing significant ADP-ribosylated epi-transcriptome dependent on PARP12. (**B**) Bubble plot represents significant pathway categories with multiple subpathway terms enriched by the identified PARP12 RNA substrates from A. Pathway enrichment was done using Ingenuity Pathway Analysis software and the Reactome database as a reference. (**C**) Foldchange of ADP-ribosylated *Ins2* transcript compared to NoCT1-NT group. (n=3, 2way ANOVA, Holm-Šídák's multiple comparisons test).

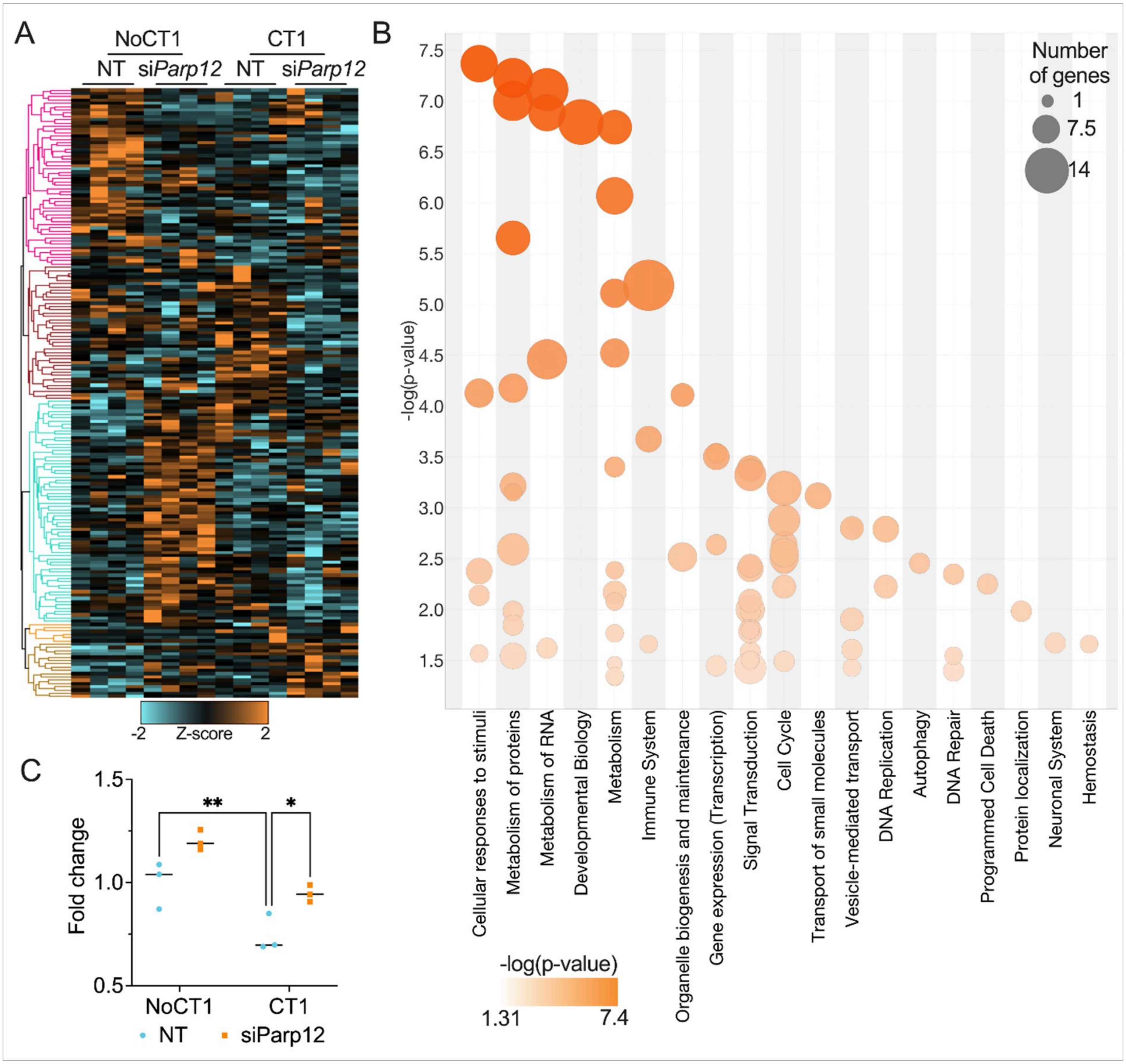

### Figure 6

Protein translational effect of PARP12. (**A**) heatmap showing significant change in abundance of newly synthesized proteins due to PARP12 knockdown identified by bottom-up proteomics post-heavy lysine pulse-chase experiment in MIN6 cells with -/+ si*Parp12* treated with CT1. (**B**) Bubble plot represents significant pathway categories enriched by PARP12-dependent proteins identified by post-heavy lysine pulse-chase experiment from A. Pathway enrichment was done by Ingenuity Pathway Analysis software, using the Reactome database as reference. (**C**) Abundance profile of nascent insulin 2 (INS2) protein.

## Supplementary Files

This is a list of supplementary files associated with this preprint. Click to download.

- ESMfile1.xlsx
